# Validation of a Deep Learning Algorithm for the Detection of Malignant Pulmonary Nodules in Chest Radiographs

**DOI:** 10.1001/jamanetworkopen.2020.17135

**Published:** 2020-09-24

**Authors:** Hyunsuk Yoo, Ki Hwan Kim, Ramandeep Singh, Subba R. Digumarthy, Mannudeep K. Kalra

**Affiliations:** 1 Lunit, Seoul, South Korea; 2 Division of Thoracic Imaging, Department of Radiology, Massachusetts General Hospital, Boston; 3 Harvard Medical School, Boston, Massachusetts

## Abstract

**Importance:**

The improvement of pulmonary nodule detection, which is a challenging task when using chest radiographs, may help to elevate the role of chest radiographs for the diagnosis of lung cancer.

**Objective:**

To assess the performance of a deep learning–based nodule detection algorithm for the detection of lung cancer on chest radiographs from participants in the National Lung Screening Trial (NLST).

**Design, Setting, and Participants:**

This diagnostic study used data from participants in the NLST ro assess the performance of a deep learning–based artificial intelligence (AI) algorithm for the detection of pulmonary nodules and lung cancer on chest radiographs using separate training (in-house) and validation (NLST) data sets. Baseline (T0) posteroanterior chest radiographs from 5485 participants (full T0 data set) were used to assess lung cancer detection performance, and a subset of 577 of these images (nodule data set) were used to assess nodule detection performance. Participants aged 55 to 74 years who currently or formerly (ie, quit within the past 15 years) smoked cigarettes for 30 pack-years or more were enrolled in the NLST at 23 US centers between August 2002 and April 2004. Information on lung cancer diagnoses was collected through December 31, 2009. Analyses were performed between August 20, 2019, and February 14, 2020.

**Exposures:**

Abnormality scores produced by the AI algorithm.

**Main Outcomes and Measures:**

The performance of an AI algorithm for the detection of lung nodules and lung cancer on radiographs, with lung cancer incidence and mortality as primary end points.

**Results:**

A total of 5485 participants (mean [SD] age, 61.7 [5.0] years; 3030 men [55.2%]) were included, with a median follow-up duration of 6.5 years (interquartile range, 6.1-6.9 years). For the nodule data set, the sensitivity and specificity of the AI algorithm for the detection of pulmonary nodules were 86.2% (95% CI, 77.8%-94.6%) and 85.0% (95% CI, 81.9%-88.1%), respectively. For the detection of all cancers, the sensitivity was 75.0% (95% CI, 62.8%-87.2%), the specificity was 83.3% (95% CI, 82.3%-84.3%), the positive predictive value was 3.8% (95% CI, 2.6%-5.0%), and the negative predictive value was 99.8% (95% CI, 99.6%-99.9%). For the detection of malignant pulmonary nodules in all images of the full T0 data set, the sensitivity was 94.1% (95% CI, 86.2%-100.0%), the specificity was 83.3% (95% CI, 82.3%-84.3%), the positive predictive value was 3.4% (95% CI, 2.2%-4.5%), and the negative predictive value was 100.0% (95% CI, 99.9%-100.0%). In digital radiographs of the nodule data set, the AI algorithm had higher sensitivity (96.0% [95% CI, 88.3%-100.0%] vs 88.0% [95% CI, 75.3%-100.0%]; *P* = .32) and higher specificity (93.2% [95% CI, 89.9%-96.5%] vs 82.8% [95% CI, 77.8%-87.8%]; *P* = .001) for nodule detection compared with the NLST radiologists. For malignant pulmonary nodule detection on digital radiographs of the full T0 data set, the sensitivity of the AI algorithm was higher (100.0% [95% CI, 100.0%-100.0%] vs 94.1% [95% CI, 82.9%-100.0%]; *P* = .32) compared with the NLST radiologists, and the specificity (90.9% [95% CI, 89.6%-92.1%] vs 91.0% [95% CI, 89.7%-92.2%]; *P* = .91), positive predictive value (8.2% [95% CI, 4.4%-11.9%] vs 7.8% [95% CI, 4.1%-11.5%]; *P* = .65), and negative predictive value (100.0% [95% CI, 100.0%-100.0%] vs 99.9% [95% CI, 99.8%-100.0%]; *P* = .32) were similar to those of NLST radiologists.

**Conclusions and Relevance:**

In this study, the AI algorithm performed better than NLST radiologists for the detection of pulmonary nodules on digital radiographs. When used as a second reader, the AI algorithm may help to detect lung cancer.

## Key Points

**Question:**

Does an artificial intelligence algorithm trained to detect pulmonary nodules improve lung cancer detection on chest radiographs?

**Findings:**

In this diagnostic study of data from 5485 participants in the National Lung Screening Trial, the sensitivity and specificity of an artificial intelligence algorithm for nodule detection were 86% and 85%, respectively. When the same artificial intelligence algorithm was applied for cancer detection, the sensitivity was 94%, specificity 83%, positive predictive value 3%, and negative predictive value was 100% for the detection of malignant pulmonary nodules.

**Meaning:**

The study findings suggest that an artificial intelligence algorithm trained to detect pulmonary nodules can help to improve lung cancer detection on chest radiographs.

## Teaser

This diagnostic study uses data from participants in the National Lung Screening Trial to assess the performance of a deep learning–based nodule detection algorithm for the detection of lung cancer on chest radiographs.

## Introduction

Large randomized clinical trials investigating chest radiography and low-dose computed tomography (CT) as screening tools for lung cancer have reported that low-dose CT screening reduces lung cancer mortality in high-risk populations.^1,2^ Therefore, only low-dose CT is recommended for lung cancer screening among high-risk populations in most countries.^3,4,5^ However, a substantial cost is associated with low-dose CT; compared with chest radiography, CT is less accessible and more expensive, exposes patients to a higher dose of radiation, and produces a higher proportion of false-positive and incidental findings, which may lead to additional laboratory testing and increase patient anxiety.^6,7^

Chest radiography avoids many of the problems associated with low-dose CT, but the survival benefits of chest radiography as a screening tool have not been fully examined.^8^ The detection of lung cancer on chest radiographs is challenging for radiologists because of the limited contrast resolution and 2-dimensional projectional nature of radiography, which can obscure findings owing to the superimposition of lesions by anatomical structures and produce high false-negative rates with low intraobserver and interobserver agreement.^9,10^ Previous studies have reported that, in retrospect, radiographic evidence of cancer was present in up to 90% of patients with peripheral cancer and in 65% to 70% of patients with centrally located cancer before the actual cancer diagnosis was made.^10,11^ Tumor characteristics, such as lesion size, conspicuity, and location, are all independent factors in detection error and can lead to missed lesions during the interpretation of chest radiographs.^12^

For these reasons, low-dose CT is the recommended modality for lung cancer screening among high-risk populations in most countries. Yet, substantial proportions of incidentally detected lung cancer cases are detected as malignant pulmonary nodules on routine chest radiographs.^13,14^ Although improved detection of lung nodules could produce overdiagnosis of low-grade cancer without decreasing mortality, patients with incidental diagnoses have better prognoses, as they are likely to have earlier stages of cancer and smaller tumors.^14^ Therefore, if errors in the detection of malignant pulmonary nodules can be reduced, more lung cancer cases that are visible but overlooked by the observer on routine chest radiographs may be detected, with subsequent survival benefits for those with detected lung cancer.

Existing studies have examined various deep learning models and techniques for abnormal image classification on chest radiographs.^15,16,17^ In the last 5 years, several artificial intelligence (AI) algorithms have been tested in an effort to decrease radiologist errors and increase the detection rate of pulmonary nodules on chest radiographs.^9,18,19,20,21^ Compared with these previous studies, the control group in our study comprised participants without disease as well as those with various types of disease, including calcified granuloma, consolidation, emphysema, and other thoracic diseases. Thus, our control group was more reflective of the wide spectrum of disease encountered in clinical practice.^9,21,22^ The goal of this study was to evaluate whether an AI algorithm trained for pulmonary nodule detection could be applied for lung cancer detection on chest radiographs from participants in the multicenter National Lung Screening Trial (NLST) to validate the generalizability and accuracy of our AI approach.^22^

## Methods

This diagnostic study assessed the performance of a deep learning–based AI algorithm for the detection of pulmonary nodules and lung cancer on chest radiographs using separate training (in-house) and validation (NLST) data sets. Ethics review and approval were obtained from the institutional review board of Massachusetts General Hospital. The need for informed consent was waived because our retrospective study used previously acquired data from other clinical trials (NLST). This study followed the Standards for Reporting of Diagnostic Accuracy (STARD) reporting guideline.

### Overview

Our retrospective analysis used data from participants in the NLST, a multicenter randomized clinical trial comparing low-dose CT with chest radiography for the screening of a high-risk population.^22,23^ From August 2002 through April 2004, eligible participants were enrolled in 33 US centers and randomized to receive either low-dose CT screening or chest radiography screening.^22^ Participants were eligible if they were aged 55 to 74 years with at least 30 pack-years of cigarette smoking; participants who formerly smoked were eligible if they had quit smoking within the previous 15 years. The participants were offered 3 annual chest radiography screenings (at T0, T1, and T2), and were followed up until December 31, 2009, for lung cancer incidence and mortality, which were the primary end points of the study. Of 9362 participants enrolled through the American College of Radiology Imaging Network and randomized to the chest radiography arm, 5485 participants (83% of whom were randomly selected) from 21 sites who had valid Digital Imaging and Communications in Medicine files (full T0 data set) were included in our analysis.

The chest radiographs comprised images from multiple vendors that were obtained using various types of chest radiography, including screen-film, computed, digital, and thoravision radiography. For each image, radiologists who participated in the NLST study annotated the type of the abnormality present, and commented on the presence of any finding suggestive of lung cancer (ie, nodule[s] ≥4 mm or enlarging nodule[s], mass[es], and other nonspecific abnormalities) that warranted further diagnostic evaluation.^22^

The radiologists who participated in the NLST had American Board of Radiology certification or its equivalent, radiologic training during residency, involvement in the supervision and interpretation of at least 300 chest CT acquisitions, performance of at least 200 chest radiography acquisitions per year, and participation in continuing medical education in accordance with American College of Radiology standards.^22^

### Preparation and Annotation

To evaluate the performance of the AI algorithm for the detection of pulmonary nodules, a subset of 577 baseline (T0) images (nodule data set) were selected and reannotated for the presence of nodules with the assistance of clinical information or follow-up imaging examinations. First, we selected chest radiographs of 48 patients who received lung cancer diagnoses within 1 year of the T0 screening. Second, we selected chest radiographs of 50 patients who received all 3 years of screening radiographs and had noncalcified nodules (as annotated by NLST radiologists) but did not have lung cancer diagnoses. Third, we selected chest radiographs of 480 patients who did not meet the first 2 criteria. To reduce selection bias, chest radiographs were selected sequentially, in order of the coded patient identification numbers. One chest radiograph that did not include an image of the entire lung was removed. In the selection process, a maximum of 1 chest radiograph was selected per patient.

The nodule data set was labeled by 2 radiologists (K.H.K., with 6 years of experience, and M.K., with 21 years of experience). Each radiologist independently evaluated T0 chest radiographs for the presence of noncalcified nodules that were 4 mm or greater on the image level. To improve the accuracy of the radiologic evaluation, each patient’s cancer characteristics, all available sequential chest radiographs, and NLST radiologist labels for the chest radiographs were provided during annotation. A chest radiograph was labeled positive for noncalcified nodules if the 2 radiologists agreed on the presence of noncalcified nodules and negative if the 2 radiologists did not agree. The final labels generated in this study are publicly available.^24^

Lung cancer was considered to be present if cancer was diagnosed during the interval from 1 chest radiography screening to the next screening or 1 year after the last chest radiography screening, whichever occurred first.^3,5^ A chest radiograph was labeled positive for malignant pulmonary nodules if it had positive findings for the presence of both lung cancer and noncalcified nodules. The 2 radiologists (K.H.K. and M.K.) retrospectively reviewed cancer cases that were not labeled malignant pulmonary nodules and recorded whether any visible radiologic findings (other than pulmonary nodules) suggestive of lung cancer were present. An image was labeled positive for other visible radiologic findings if the 2 radiologists agreed on the presence of the findings.

### Algorithm for Analysis

We used a commercially available AI algorithm (Lunit INSIGHT CXR) to analyze the initial chest radiographs. The AI algorithm we selected is a deep convolutional neural network that uses residual neural network 34 (ResNet-34) as its foundational architecture.^25^ In the preprocessing step, the raw pixel map of the Digital Imaging and Communications in Medicine file was normalized with windowing information, and the normalized pixel map was used as input for the AI model. The model then produced a coarse probability map with multiple channels, with an array value that defined the probability of the target lesions being present, and was generated using a weakly supervised object localization technique.^26^ Each channel defined the probability map for each target lesion, and the probability map was used to localize possible abnormal regions for the target lesion. The image-level probability score was then max pooled (a technique that reduces the dimensionality of images by reducing the number of pixels in the output from the previous convolutional layer) from the output probability map to generate a final abnormality score between 0 and 100. The commercial AI algorithm produced a probability map and score for 10 abnormalities; however, only output corresponding to noncalcified nodules was used in this study.

During training of the model, we used augmentation policies from AutoAugment (Google Brain), which defines information about which image processing operations to use, such as translation, rotation, or shearing, and the magnitude of the operations.^27^ When image-level labels were available, binary cross-entropy loss was computed using image-level probability scores and image-level labels; when ground truth annotation maps (ie, pixelwise lesion contour–label maps used for supervised training) were available, the binary cross-entropy loss was computed using both probability maps and annotation maps.

The training set included 12 408 abnormal images with lung nodules or masses that were obtained from patients with pathologically proven and/or radiologically confirmed diseases, which were verified by at least 1 of 15 board-certified radiologists (with 7-14 years of experience), and 72 704 normal images, all of which were obtained from multiple hospitals in South Korea.^28^ The training images included both digital and computed radiographs, and none of the NLST data were used during the training of the model. A more detailed description of the data collection process and the development of the AI algorithm can be found in a previous study.^9^

### Assessment of Performance

Images analyzed by the AI algorithm were considered positive if the abnormality score produced by the AI algorithm for the entire image was higher than the operating point. The operating point was used to classify the presence of noncalcified nodules at the image level. In the nodule data set, both malignant and benign noncalcified nodules were labeled positive, and detection of either type of nodule was regarded as true positive. In our analysis, the operating point of the AI model was set at 15.0, which was chosen using Youden criteria in the internal validation set. At this operating point, the AI model had sensitivity of 96.6% and specificity of 94.1% for the classification of abnormal images in the internal validation set. To assess the performance of NLST radiologists, we used 2 labels provided in the NLST data set: the nodule label, which recorded the presence of noncalcified nodules or masses, and the cancer label, which recorded nodule(s) of 4 mm or greater or enlarging nodule(s), mass(es), or other nonspecific abnormalities suggestive of lung cancer.

The change in the performance of the AI algorithm compared with the performance of the NLST radiologists for the detection of nodules, lung cancer, and malignant pulmonary nodules was analyzed on balanced test data sets. In the nodule data set, nonnodules were randomly selected to have 1:1, 1:2, and 1:3 nodule to nonnodule ratios. In the full T0 data set, noncancer and nonmalignant pulmonary nodules were randomly selected to have 1:1, 1:2, and 1:3 cancer to noncancer ratios and malignant pulmonary nodule to nonmalignant pulmonary nodule ratios, respectively. Nodule labels were used to assess NLST radiologist performance for nodule detection; cancer labels were used for cancer and malignant nodule detection.

### Statistical Analysis

Receiver operating characteristic (ROC) analyses were performed to evaluate the classification performance of the AI algorithm in the nodule data set. For comparison of the sensitivities and specificities of the AI algorithm vs the NLST radiologists, the McNemar test was used. For comparison of positive predictive values (PPVs) and negative predictive values (NPVs) of the AI algorithm vs NLST radiologists, the generalized score statistic was used.^29^ The performance of the AI algorithm vs the NLST radiologists was compared in all radiographs, digital radiographs, and computed radiographs. The agreement for noncalcified nodule annotation between each ground truth annotator and the NLST radiologists was assessed with weighted κ using linear weighting. We computed 95% CIs for the performance and agreement measures with 10 000 bootstrap replications. For all tests, *P* < .05 was considered statistically significant. All statistical analyses were conducted using R software, version 3.6.1 (R Foundation for Statistical Computing). Analyses were performed between August 20, 2019, and February 14, 2020.

## Results

After excluding 1 participant with only a lateral chest radiograph and 5 participants with corrupted Digital Imaging and Communications in Medicine files, a total of 5485 patients (mean [SD] age, 61.7 [5.0] years; 3030 men [55.2%]; median follow-up duration, 6.5 years [interquartile range, 6.1-6.9 years]) were included. Of the 5485 participants with valid T0 chest radiographs selected for further analysis, 48 patients (0.9%) received a diagnosis of lung cancer within 1 year of the baseline imaging. A subset of 577 participants was selected for the nodule data set. The demographic characteristics of participants with valid T0 posteroanterior images and participants selected for the nodule data set are shown in Table 1.

**Table 1.** Participant Characteristics

Characteristic	No. (%)	
	Full T0 data set	Nodule data set
Total participants, No.	5485	577
Participants with cancer	48 (0.9)	48 (8.3)
Age, mean (SD)	61.7 (5.0)	62.2 (5.1)
Sex		
Male	3030 (55.2)	322 (55.8)
Female	2455 (44.8)	255 (44.2)
Race		
White	5145 (93.8)	539 (93.4)
Black or African American	222 (4.0)	22 (3.8)
Asian	39 (0.7)	5 (0.9)
American Indian or Alaskan Native	17 (0.3)	3 (0.5)
Native Hawaiian or other Pacific Islander	1 (0.02)	0
>1 race	36 (0.7)	6 (1.0)
Unavailable	25 (0.5)	2 (0.4)
Ethnicity		
Hispanic or Latino	77 (1.4)	5 (0.9)
Not Hispanic or Latino	5385 (98.2)	572 (99.1)
Unavailable	23 (0.4)	0
Smoking status		
Former	2765 (50.4)	306 (53.0)
Current	2720 (49.6)	271 (47.0)
Type of chest radiography used		
Screen film	47 (0.9)	4 (0.7)
Computed	2861 (52.2)	296 (51.3)
Digital	2108 (38.4)	246 (42.6)
Thoravision	465 (8.5)	31 (5.4)
Unavailable	4 (0.07)	0
Outcomes		
Follow-up, median (IQR), y	6.5 (6.1-6.9)	6.5 (6.1-6.8)
Mortality	380 (6.9)	53 (9.2)

Abbreviations: IQR, interquartile range; T0, baseline.

Of the 48 participants who received a cancer diagnosis within 1 year of baseline imaging, 34 participants had visible malignant nodules present in their chest radiographs. Among the other 14 participants, 11 participants had no visible lesions present in their chest radiographs, and 3 participants had other radiologic manifestations that were suggestive of lung cancer, including atelectasis (n = 1), pleural thickening (n = 1), and hilar lymphadenopathy (n = 1).

The agreement between each ground truth annotator and the NLST radiologists for noncalcified nodule annotation was assessed. Moderate agreement was observed between each ground truth annotator (κ = 0.55; 95% CI, 0.47-0.64) and the NLST radiologists (κ = 0.60; 95% CI, 0.52-0.69).

### Nodule Detection Performance

**Figure 1.**
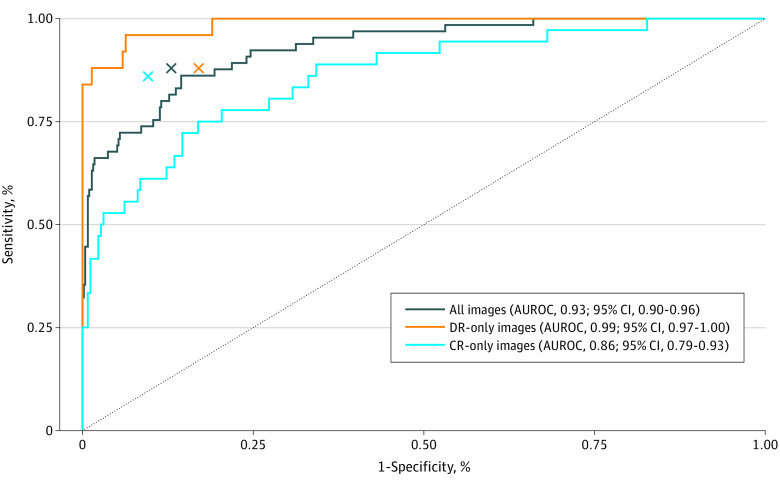
Receiver Operating Characteristic Curve of the Performance of the Artificial Intelligence Algorithm vs NLST Radiologists for the Detection of Noncalcified Nodules in the Nodule Data Set Colored lines represent results from the artificial intelligence algorithm, and colored Xs represent results from NLST radiologists. AUROC indicates area under the receiver operating characteristic; CR, computed radiography; DR, digital radiography; and NLST, National Lung Screening Trial.

The performance of the AI algorithm vs the NLST radiologists for the detection of noncalcified nodules in the nodule data set was assessed. The area under the ROC curve (AUROC) of the AI algorithm was 0.93 (95% CI, 0.90-0.96) for all chest radiographs, 0.99 (95% CI, 0.97-1.00) for digital radiographs, and 0.86 (95% CI, 0.79-0.93) for computed radiographs (Figure 1). The sensitivity and specificity pairs of the NLST radiologists (indicated by Xs in Figure 1) were under the ROC curve for digital radiographs and above the ROC curve for computed radiographs.

The differences between the AI algorithm and the NLST radiologists in both sensitivity (86.2% [95% CI, 77.8%-94.6%] vs 87.7% [95% CI, 79.7%-95.7%], respectively; *P* = .80) and specificity (85.0% [95% CI, 81.9%-88.1%] vs 86.7% [83.8%-89.7%]; *P* = .42) were statistically nonsignificant in all chest radiographs at the operating point chosen from the internal validation set. The nodule detection performance at other operating points is shown in eFigure 1 in the Supplement.

The sensitivity and specificity of the AI algorithm were higher compared with those of the NLST radiologists for digital radiographs (for sensitivity, 96.0% [95% CI, 88.3%-100.0%] vs 88.0% [95% CI, 75.3%-100.0%], respectively; *P* = .32; for specificity, 93.2% [95% CI, 89.9%-96.5%] vs 82.8% [95% CI, 77.8%-87.8%]; *P* = .001) but were lower compared with those of the NLST radiologists for computed radiographs (for sensitivity, 77.8% [95% CI, 64.2%-91.4%] vs 86.1% [95% CI, 74.8%-97.4%]; *P* = .37; for specificity, 78.8% [95% CI, 73.9%-83.8%] vs 90.4% [95% CI, 86.8%-94.0%]; *P* < .001). Of the 65 total noncalcified nodules or masses present in the nodule data set, 56 nodules or masses were detected by the AI algorithm (including 7 nodules or masses that were missed by NLST radiologists), 57 nodules or masses were detected by NLST radiologists (including 8 nodules or masses that were missed by the AI algorithm), 49 nodules or masses were detected by both, and 1 nodule or mass was missed by both.

### Cancer Detection Performance

The performance of the AI algorithm compared with the NLST radiologists for the detection of all cancers and malignant pulmonary nodules in the nodule data set and the full T0 data set is shown in Table 2 and eTable 1 in the Supplement. The sensitivity, specificity, PPV, and NPV of the AI algorithm were 75.0% (95% CI, 62.8%-87.2%), 83.3% (95% CI, 82.3%-84.3%), 3.8% (95% CI, 2.6%-5.0%), and 99.8% (95% CI, 99.6%-99.9%) for the detection of all cancers in all chest radiographs of the full T0 data set. In digital radiographs of the full T0 data set, the AI algorithm and the NLST radiologists (as assessed by the cancer label) had similar sensitivity (76.0% [95% CI, 59.3%-92.7%] vs 80.0% [95% CI, 64.3%-95.7%], respectively; *P* = .65), similar specificity (90.0% [95% CI, 89.7%-92.2%] vs 91.1% [95% CI, 89.9%-92.3%]; *P* = .82), similar PPV (9.1% [95% CI, 5.2%-13.0%] vs 9.8% [95% CI, 5.7%-13.8%]; *P* = .62), and similar NPV (99.7% [95% CI, 99.4%-99.9%] vs 99.7% [95% CI, 99.5%-100.0%]; *P* = .65) for cancer detection. In computed radiographs of the full T0 data set, the AI algorithm had lower sensitivity (68.4% [95% CI, 47.5%-89.3%] vs 89.5% [95% CI, 75.7%-100.0%]; *P* = .10), lower specificity (76.7% [95% CI, 75.2%-78.3%] vs 91.4% [95% CI, 90.3%-92.4%]; *P* < .001), lower PPV (1.9% [95% CI, 0.9%-3.0%] vs 6.3% [95% CI, 3.5%-9.5%]; *P* < .001), and similar NPV (99.7% [95% CI, 99.5%-99.9%] vs 99.9% [95% CI, 99.8%-100.0%]; *P* = .07) compared with the NLST radiologists. Among all images of the 48 participants who received lung cancer diagnoses within 1 year of screening, 36 cases were detected by the AI algorithm, 41 cases were detected by the NLST radiologists, 33 cases were detected by both, and 4 cases were missed by both.

**Table 2.** Comparison of Performance of Artificial Intelligence Algorithm vs National Lung Screening Trial Radiologists

Variable	All images					Digital radiographic images					Computed radiographic images				
	AI	NLST nodule	NLST cancer	P value		AI	NLST nodule	NLST cancer	P value		AI	NLST nodule	NLST cancer	P value	
				AI vs NLST nodule	AI vs NLST cancer				AI vs NLST nodule	AI vs NLST cancer				AI vs NLST nodule	AI vs NLST cancer
Sensitivity (all cancer detection)															
Nodule data set	75.0 (62.8-87.2)	77.1 (65.2-89.0)	85.4 (75.4-95.4)	.78	.13	76.0 (59.3-92.7)	68.0 (49.7-86.3)	80.0 (64.3-95.7)	.41	.65	68.4 (47.5-89.3)	84.2 (67.8-100.0)	89.5 (75.7-100.0)	.26	.10
Full T0 data set	75.0 (62.8-87.2)	77.1 (65.2-89.0)	85.4 (75.4-95.4)	.78	.13	76.0 (59.3-92.7)	68.0 (49.7-86.3)	80.0 (64.3-95.7)	.41	.65	68.4 (47.5-89.3)	84.2 (47.5-89.3)	89.5 (75.7-100.0)	.26	.10
Specificity (all cancer detection)															
Nodule data set	81.7 (78.4-85.0)	83.4 (80.2-86.5)	83.9 (80.8-87.1)	.43	.30	91.0 (87.2-94.7)	80.5 (75.3-85.8)	82.4 (77.3-87.4)	.001	.009	74.7 (69.6-79.8)	85.6 (81.4-89.7)	85.2 (81.0-89.4)	<.001	<.001
Full T0 data set	83.3 (82.3-84.3)	91.2 (90.4-91.9)	91.5 (90.7-92.2)	<.001	<.001	90.0 (89.7-92.2)	90.4 (89.1-91.7)	91.1 (89.9-92.3)	.52	.82	76.7 (75.2-78.3)	91.6 (90.5-92.6)	91.4 (90.3-92.4)	<.001	<.001
Sensitivity (malignant pulmonary nodule detection)															
Nodule data set	94.1 (86.2-100.0)	91.2 (81.6-100.0)	94.1 (86.2-100.0)	.65	>.99	100.0 (100.0-100.0)	88.2 (72.9-100.0)	94.1 (82.9-100.0)	.16	.32	85.7 (67.4-100.0)	92.9 (79.4-100.0)	92.9 (79.4-100.0)	.56	.56
Full T0 data set	94.1 (86.2-100.0)	91.2 (81.6-100.0)	94.1 (86.2-100.0)	.65	>.99	100.0 (100.0-100.0)	88.2 (72.9-100.0)	94.1 (82.0-100.0)	.16	.32	85.7 (67.4-100.0)	92.9 (79.4-100.0)	92.9 (79.4-100.0)	.56	.56
Specificity (malignant pulmonary nodule detection)															
Nodule data set	81.4 (78.1-84.7)	82.7 (79.5-85.9)	82.7 (79.5-85.9)	.56	.56	90.4 (86.6-94.2)	80.3 (75.2-85.5)	81.2 (76.2-86.3)	.002	.005	74.8 (69.8-79.9)	84.8 (80.6-88.9)	84.0 (79.8-88.3)	.002	.003
Full T0 data set	83.3 (82.3-84.3)	91.1 (90.3-91.8)	91.3 (90.6-92.1)	<.001	<.001	90.9 (89.6-92.1)	90.3 (89.1-91.6)	91.0 (89.7-92.2)	.53	.91	76.7 (75.2-78.3)	91.5 (90.4-92.5)	91.3 (90.2-92.3)	<.001	<.001

Abbreviations: AI, artificial intelligence; NLST, National Lung Screening Trial; NLST cancer, National Lung Screening Trial radiologists using cancer label; NLST nodule, National Lung Screening Trial radiologists using nodule label; T0, baseline.

In all radiographs of the full T0 data set, the sensitivity, specificity, PPV, and NPV of the AI algorithm were 94.1% (95% CI, 86.2%-100.0%), 83.3% (95% CI, 82.3%-84.3%), 3.4% (95% CI, 2.2%-4.5%), and 100.0% (95% CI, 99.9%-100.0%), respectively, for the detection of malignant pulmonary nodules. In digital radiographs of the full T0 data set, the AI algorithm had higher sensitivity (100.0% [95% CI, 100.0%-100.0%] vs 94.1% [95% CI, 82.9%-100.0%]; *P* = .32), similar specificity (90.9% [95% CI, 89.6%-92.1%] vs 91.0% [95% CI, 89.7%-92.2%]; *P* = .91), similar PPV (8.2% [95% CI, 4.4%-11.9%] vs 7.8% [95% CI, 4.1%-11.5%]; *P* = .65), and similar NPV (100.0% [95% CI, 100.0%-100.0%] vs 99.9% [95% CI, 99.8%-100.0%]; *P* = .32) compared with the NLST radiologists (as assessed by the cancer label). In computed radiographs of the full T0 data set, the AI algorithm had lower sensitivity (85.7% [95% CI, 67.4%-100.0%] vs 92.9% [95% CI, 79.4%-100.0%]; *P* = .56), lower specificity (76.7% [95% CI, 75.2%-78.3%] vs 91.3% [95% CI, 90.2%-92.3%]; *P* < .001), lower PPV (1.8% [95% CI, 0.8%-2.8%] vs 5.0% [95% CI, 2.3%-7.6%]; *P* < .001), and similar NPV (99.9% [95% CI, 99.8%-100.0%] vs 100.0% [95% CI, 99.9%-100.0%]; *P* = .48) compared with the NLST radiologists. In all images of the 34 patients with malignant pulmonary nodules who received lung cancer diagnoses within 1 year after imaging, 32 cases were detected by the AI algorithm, 32 cases were detected by the NLST radiologists, 30 cases were detected by both, and 0 cases were missed by both.

The performance of the AI algorithm (as measured by AUROC, sensitivity, and specificity) and the performance of the NLST radiologists (as measured by sensitivity and specificity) remained consistent at different ratios (1:1, 1:2, and 1:3) of control images for all tasks (eTable 2 in the Supplement). The small variations in the performance of the AI algorithm and the NLST radiologists at different control group ratios were all within the 95% CIs of the performance in the nodule data set and the full T0 data set for all tasks.

## Discussion

In this study, we applied an AI algorithm, which was originally trained for pulmonary nodule detection, for the detection of lung cancer on chest radiographs. In the nodule data set, the sensitivity and specificity of the AI algorithm for nodule detection were 86.2% and 85.0%, respectively. When the same AI algorithm was applied for cancer detection, the sensitivity (76.0% vs 80.0%) and PPV (9.1% vs 9.8%) of the AI algorithm were similar to those of the NLST radiologists for the detection of all cancers. In digital radiographs of the full T0 data set, the sensitivity (100.0% vs 94.1%) and PPV (8.2% vs 7.8%) were also similar to those of the NLST radiologists for the detection of malignant pulmonary nodules. We also observed that the performance of the AI algorithm remained consistent even with changes in the ratio of control images. Although the sensitivity of the AI algorithm for lung cancer detection in this study is lower than the sensitivity reported for low-dose CT (93.8%), it is similar to the reported sensitivity of radiologists for chest radiographs (73.5%).^3^

The nodule data set used in this study included chest radiographs of both benign and malignant nodules as well as chest radiographs of other abnormalities, such as consolidation and emphysema. The data set was designed to reflect the distribution of chest radiographs that may be encountered in a cancer screening setting. Despite our nodule data set design, the AI algorithm retained high performance for nodule detection. In our study, the overall AUROC for nodule detection was 0.93 (95% CI, 0.90-0.96), which is similar to the AUROCs of 0.92 to 0.99 reported by Nam et al^9^ and the AUROC of 0.91 reported by Majkowska et al^30^ in a study of data from the National Institutes of Health Chest x-ray Data set of 14 Common Thorax Disease Categories.

Notably, we also observed that the AI algorithm performed better in digital radiographs (AUROC, 0.99) than in computed radiographs (AUROC, 0.86). Although the data used to train the AI model included both digital and computed radiographs, the inferior quality of the older computed radiographs included in the nodule data set may have been unencountered during training, which would account for the decreased performance of the AI algorithm in computed radiographs. As a consequence, the AI model could not equal the performance of the NLST radiologists for the detection of noncalcified nodules in computed radiographs.

In this study, the AI algorithm had lower PPV and similar NPV compared with the NLST radiologists for the detection of all cancers and malignant pulmonary nodules. In older computed radiographs, the AI algorithm frequently provided false-positive readings, which produced low PPV. However, on the more contemporary digital radiographs, the AI algorithm performed better than the NLST radiologists with regard to sensitivity and had a PPV similar to that of the NLST radiologists for the detection of all cancers and malignant pulmonary nodules. This finding suggests that the AI results should be interpreted with caution when the algorithm is performed on a test set with characteristics different from those of the training set.

One of the most important contributions of our research is its assessment of the performance of an AI algorithm that was originally designed to detect pulmonary nodules for the detection of cancer using a data set from the NLST, the multicenter randomized clinical trial on which the selection criteria for current lung cancer screenings via imaging are based. Although the prevalence of lung cancer may be higher in the NLST data set compared with the general population, the test set included a spectrum of diseases that may be encountered in clinical practice.

There was a moderate decrease in the detection performance of the AI algorithm when it was applied for the detection of any lung cancer, but the AI algorithm had high performance for the detection of malignant pulmonary nodules. Retrospective radiologic assessment of all lung cancer cases in the full T0 data set indicated that only 34 of 48 all-cancer cases presented as malignant nodules. Of the remaining 14 cases, 11 cases had no visible lesions, and 3 cases had other radiologic manifestations, such as hilar lymphadenopathy, pleural abnormalities, and sublobar atelectasis, as described in the literature.^31^ Because the AI algorithm used in this study is designed to specifically detect lung nodules or masses, the algorithm likely missed lung cancer cases that did not present as malignant pulmonary nodules.

In the digital radiographs of the full T0 data set, the sensitivity of the AI algorithm for malignant pulmonary nodule detection was greater than that of the NLST radiologists, as assessed by both nodule and cancer labels. In addition, although the AI model had lower sensitivity compared with the NLST radiologists for the detection of all cancers, the sensitivity remained greater than that of the NLST radiologists, as assessed by the nodule label. Such results suggest that the AI algorithm has excellent sensitivity for detecting not only noncalcified nodules but also malignant pulmonary nodules, even performing better than radiologists.

**Figure 2.**
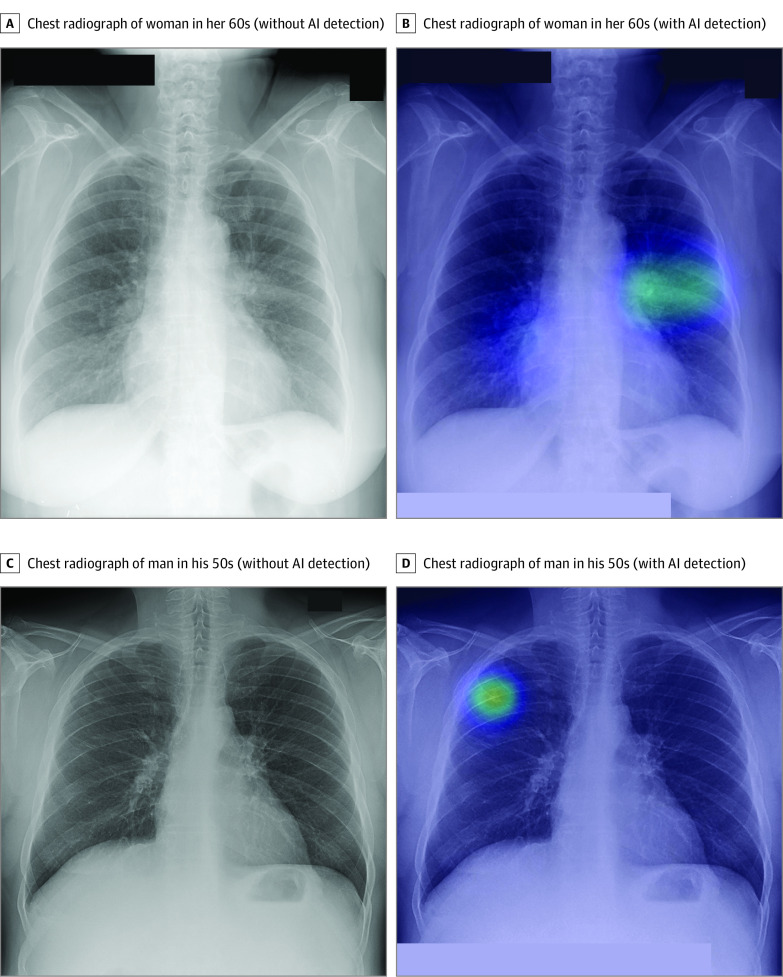
Frontal Chest Radiographs of Patients With Malignant Pulmonary Nodules Missed by NLST Radiologists But Detected by Artificial Intelligence Algorithm A, Chest radiograph of woman in her 60s (without AI detection). The woman was diagnosed with lung cancer 86 days after baseline imaging. B, Chest radiograph of woman in her 60s (with AI detection). The AI algorithm detected the missed subtle abnormality (in green, with nodule score of 38%) in the left perihilar region. C, Chest radiograph of man in his 50s (without AI detection). The man was diagnosed with lung cancer 127 days after baseline imaging. D, Chest radiograph of man in his 50s (with AI detection). The AI algorithm detected the missed subcentimeter nodule (in green, with nodule score of 53%) in the right upper lung zone. AI indicates artificial intelligence; and NLST, National Lung Screening Trial.

In this study, modest agreement was observed between the NLST radiologists and the ground truth annotators. Given the interreader variability of pulmonary nodule detection in chest radiographs, we believe that the likelihood of mislabeling by radiologists will be high in instances when only a single reader interprets a chest radiograph. A recent study by Majkowska et al^30^ indicated that a substantial number of nonoverlapping true-positive findings exist for various types of lesions detected by an AI algorithm and radiologists. This finding is consistent with that of our study, which found that several noncalcified nodules and malignant pulmonary nodules that were missed by radiologists were detected only by the AI algorithm and vice versa. Of the 65 total noncalcified nodules or masses present in the nodule data set, 8 nodules or masses were missed by the NLST radiologists, 7 of which were also detected only by the AI algorithm. In addition, of the 34 total malignant pulmonary nodules, 2 nodules missed by the NLST radiologists were detected only by the AI algorithm (Figure 2). These findings underscore the value of an AI algorithm as a second reader during the interpretation of chest radiographs for lung nodules.

### Limitations

Our study has several limitations. First, although NLST included a community cohort of participants with a high risk of lung cancer, the prevalence of lung cancer remained low, and only 48 participants with lung cancer were available for inclusion in the present analysis. Because of the small number of participants with cancer, it was difficult to achieve statistical significance for the differences in sensitivity between the AI algorithm and the NLST radiologists. Second, because NLST data were collected between 2002 and 2004, the quality of the chest radiographs might have been inferior to those obtained using modern equipment; this inferiority may have led to underperformance of the AI algorithm (particularly for computed radiographs), which was trained on chest radiographs that were all obtained after 2010. The decrease in performance was especially pronounced for computed radiographs; the 2 malignant nodules missed by the AI algorithm but detected by the NLST radiologists were both from computed radiographs (eFigure 2 in the Supplement).

Third, the term NLST radiologist was used to refer to a pool of radiologists who participated in the NLST, and the actual performance of individual radiologists may be different from the pooled performance of NLST radiologists. Fourth, ground truth labels of nodules were generated without paired CT images; thus, the labels may have been inaccurate. Fifth, we did not assess the incremental value of the AI algorithm as a second reader by conducting a prospective study that comprised separate sets of radiologists, who may have had different performance than the NLST radiologists. Sixth, chest radiographs are no longer recommended for lung cancer screening; therefore, the results of this study might not be implemented for lung cancer screening.

## Conclusions

The AI algorithm performed better than the NLST radiologists for the detection of all noncalcified nodules and malignant pulmonary nodules on digital radiographs. The AI algorithm may help to detect lung cancer by detecting additional malignant pulmonary nodules.

## Notes

Supplement. **eTable 1.** Comparison of Positive Predictive Values and Negative Predictive Values of AI Algorithm vs NLST Radiologists in Full T0 Data Set **eTable 2.** Performance of AI Algorithm vs NLST Radiologists in Balanced Test Set **eFigure 1.** Nodule Detection Performance at Various Operating Points in the Nodule Data Set **eFigure 2.** Frontal Chest Radiographs of Patients With Malignant Pulmonary Nodules That Were Found by NLST Radiologists but Missed by the AI Algorithm
