# Supplementary material for: Validation of a Deep Learning Algorithm for the Detection of Malignant Pulmonary Nodules in Chest Radiographs
Source: JAMA Netw Open. 2020 Sep 24;3(9):e2017135. doi: 10.1001/jamanetworkopen.2020.17135 (PMC7516603; doi:10.1001/jamanetworkopen.2020.17135)
Supplement: Supplement. — eTable 1. Comparison of Positive Predictive Values and Negative Predictive Values of AI Algorithm vs NLST Radiologists in Full T0 Data Set eTable 2. Performance of AI Algorithm vs NLST Radiologists in Balanced Test Set eFigure 1. Nodule Detection Performance at Various Operating Points in the Nodule Data Set eFigure 2. Frontal Chest Radiographs of Patients With Malignant Pulmonary Nodules That Were Found by NLST Radiologists but Missed by the AI Algorithm [file jamanetwopen-e2017135-s001.pdf]

Supplementary Online Content

Yoo H, Kim KH, Singh R, Digumarthy SR, Kalra MK. Validation of a deep learning algorithm for the detection of malignant pulmonary nodules in chest radiographs. *JAMA Netw Open*. 2020;3(9):e2017135. doi:10.1001/jamanetworkopen.2020.17135

eTable 1. Comparison of Positive Predictive Values and Negative Predictive Values of AI Algorithm vs NLST Radiologists in Full T0 Data Set

eTable 2. Performance of AI Algorithm vs NLST Radiologists in Balanced Test Set

eFigure 1. Nodule Detection Performance at Various Operating Points in the Nodule Data Set

eFigure 2. Frontal Chest Radiographs of Patients With Malignant Pulmonary Nodules That Were Found by NLST Radiologists but Missed by the AI Algorithm

This supplementary material has been provided by the authors to give readers additional information about their work.

© 2020 Yoo H et al. *JAMA Network Open*.

eTable 1. Comparison of Positive Predictive Values and Negative Predictive Values of AI Algorithm vs NLST Radiologists in Full T0 Data Set

Comparison of the performance of AI and NLST radiologists for the detection of all lung cancer and malignant pulmonary nodules in full T0 data set. PPV and NPV were compared in the nodule data set (all image: n=577, DR images: n=246, CR images: n=296) and full T0 data set (all image: n=5485, DR images: n=2108, CR images: n=2861). NLST-Nodule = NLST radiologists using the nodule label. NLST-Cancer = NLST radiologists using the cancer label.

	**All images**					**DR**					**CR**				
	AI	NLST-Nodule	NLST-Cancer	P value (AI vs. NLST-Nodule)	P value (AI vs. NLST-Cancer)	AI	NLST-Nodule	NLST-Cancer	P value (AI vs. NLST-Nodule)	P value (AI vs. NLST-Cancer)	AI	NLST-Nodule	NLST-Cancer	P value (AI vs. NLST-Nodule)	P value (AI vs. NLST-Cancer)
**PPV (all cancer detection)**															
Full T0 Data Set	**3.8 (2.6-5.0)**	**7.1 (4.9-9.4)**	**8.1 (5.7-10.5)**	**<0.001**	**<0.001**	9.1 (5.2-13.0)	7.8 (4.3-11.4)	9.8 (5.7-13.8)	0.30	0.62	**1.9 (0.9-3.0)**	**6.2 (3.3-9.2)**	**6.3 (3.5-9.5)**	**<0.001**	**<0.001**
**NPV (all cancer detection)**															
Full T0 Data Set	99.8 (99.6-99.9)	99.8 (99.6-99.9)	99.9 (99.8-100.0)	0.58	0.08	99.7 (99.4-99.9)	99.6 (99.3-99.9)	99.7 (99.5-100.0)	0.41	0.65	99.7 (99.5-99.9)	99.9 (99.8-100.0)	99.9 (99.8-100.0)	0.17	0.07
**PPV (malignant pulmonary nodule detection)**															
Full T0 Data Set	**3.4 (2.2-4.5)**	**6.0 (3.9-8.0)**	**6.3 (4.2-8.5)**	**<0.001**	**<0.001**	8.2 (4.4-11.9)	6.9 (3.5-10.3)	7.8 (4.1-11.5)	0.15	0.65	**1.8 (0.8-2.8)**	**5.1 (2.4-7.8)**	**5.0 (2.3-7.6)**	**<0.001**	**<0.001**
**NPV (malignant pulmonary nodule detection)**															
Full T0 Data Set	100.0 (99.9-100.0)	99.9 (99.9-100.0)	100.0 (99.9-100.0)	0.73	0.93	100.0 (100.0-100.0)	99.9 (99.7-100.0)	99.9 (99.8-100.0)	0.16	0.32	99.9 (99.8-100.0)	100.0 (99.9-100.0)	100.0 (99.9-100.0)	0.48	0.48

© 2020 Yoo H et al. *JAMA Network Open*.

eTable 2. Performance of AI Algorithm vs NLST Radiologists in Balanced Test Set

The performance of AI and NLST radiologists for (a) nodule, (b) cancer and (c) malignant nodule detection in test sets that contain different ratio of control images.

(a) Performance of AI and NLST radiologists for nodule detection in test sets that contain different ratio of control images. Number of nodules in all test sets was 65. AUROC = area under the ROC curve. Sens = sensitivity. Spec = specificity.

Ratio (ndl: non-ndl)	AI AUROC	AI Sens	AI Spec	NLST Sens	NLST Spec
65:65	0.93 (0.89-0.97)	86.2 (77.8-94.6)	84.6 (75.8-93.4)	87.7 (79.7-95.7)	86.2 (77.8-94.6)
65:130	0.92 (0.89-0.96)	86.2 (77.8-94.6)	83.1 (76.6-89.5)	87.7 (79.7-95.7)	86.0 (81.1-92.7)
65:195	0.93 (0.89-0.97)	86.2 (77.8-94.6)	86.2 (81.3-91.0)	87.7 (79.7-95.7)	86.7 (81.9-91.4)
65:512 (="Nodule Data Set")	0.93 (0.90-0.96)	86.2 (77.8-94.6)	85.0 (81.9-88.1)	87.7 (79.7-95.7)	86.7 (83.8-89.7)

(b) Performance of AI and NLST radiologists for cancer detection in test sets that contain different ratio of control images. Number of lung cancer in all test sets was 48. AUROC = area under the ROC curve. Sens = sensitivity. Spec = specificity.

Ratio (ndl: non-ndl)	AI AUROC	AI Sens	AI Spec	NLST Sens	NLST Spec
48:48	0.84 (0.76-0.93)	75.0 (62.8-87.2)	81.2 (70.2-92.3)	85.4 (75.4-95.4)	93.8 (86.9-100.0)
48:96	0.87 (0.80-0.95)	75.0 (62.8-87.2)	88.5 (82.2-94.9)	85.4 (75.4-95.4)	91.7 (86.1-97.2)
48:144	0.86 (0.78-0.93)	75.0 (62.8-87.2)	81.9 (75.7-88.2)	85.4 (75.4-95.4)	91.0 (86.3-95.7)
48:5437 (="Full T0 Data Set")	0.85 (0.77-0.93)	75.0 (62.8-87.2)	83.3 (82.3-84.3)	85.4 (75.4-95.4)	91.5 (90.7-92.2)

© 2020 Yoo H et al. *JAMA Network Open*.

(c) Performance of AI and NLST radiologists for malignant pulmonary nodule detection in test sets that contain different ratio of control images. Number of malignant pulmonary nodules in all test sets was 34. AUROC = area under the ROC curve. Sens = sensitivity. Spec = specificity.

Ratio (ndl: non-ndl)	AI AUROC	AI Sens	AI Spec	NLST Sens	NLST Spec
34:34	0.97 (0.93-1.00)	94.1 (86.2-100.0)	85.3 (73.4-97.2)	94.1 (86.2-100.0)	88.2 (77.4-99.1)
34:68	0.96 (0.93-1.00)	94.1 (86.2-100.0)	85.3 (76.9-93.7)	94.1 (86.2-100.0)	91.2 (84.4-97.9)
34:102	0.97 (0.94-1.00)	94.1 (86.2-100.0)	80.4 (72.7-88.1)	94.1 (86.2-100.0)	85.3 (78.4-92.2)
34:5451 (="Full T0 Data Set")	0.97 (0.94-1.00)	94.1 (86.2-100.0)	83.3 (82.3-84.3)	94.1 (86.2-100.0)	93.1 (90.6-92.1)

© 2020 Yoo H et al. *JAMA Network Open*.

eFigure 1. Nodule Detection Performance at Various Operating Points in the Nodule Data Set

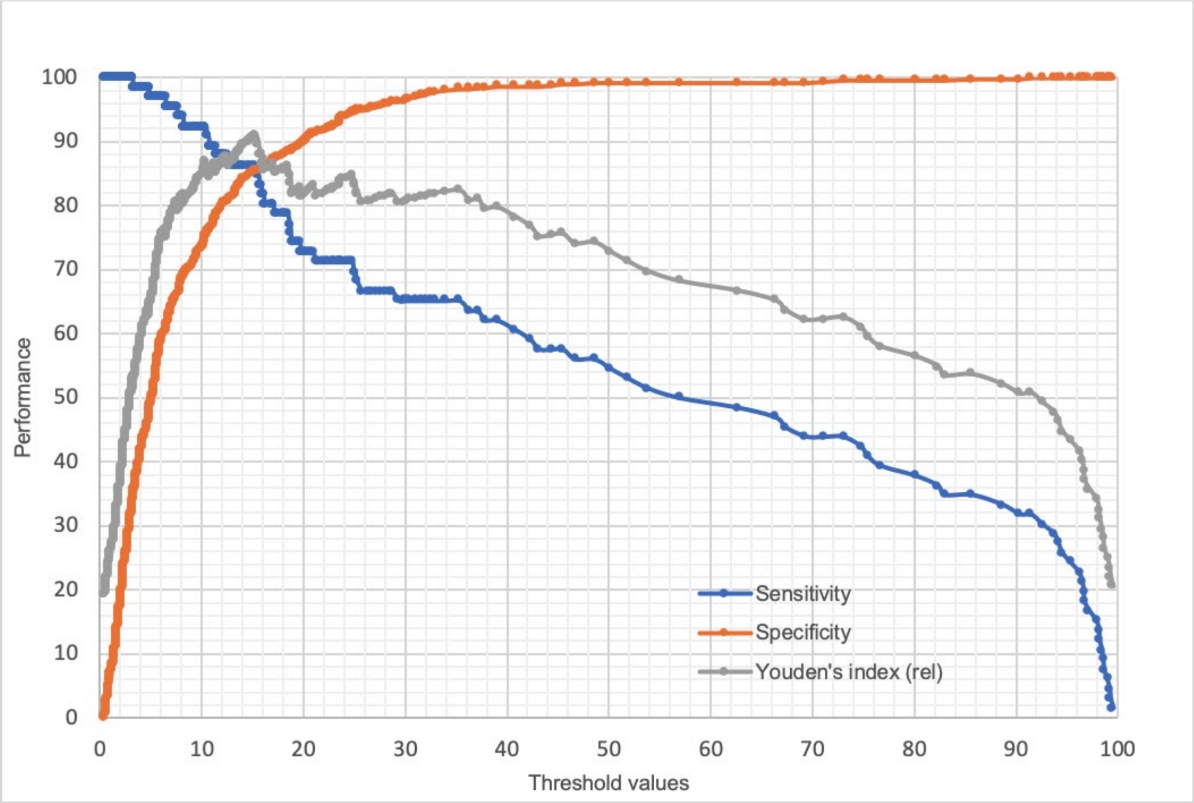

The operating point chosen from the internal validation set (=15.0) using Youden's index was close to the threshold at which the model had highest Youden's index (=15.3) in the external nodule data set.

© 2020 Yoo H et al. *JAMA Network Open*.

eFigure 2. Frontal Chest Radiographs of Patients With Malignant Pulmonary Nodules That Were Found by NLST Radiologists but Missed by the AI Algorithm

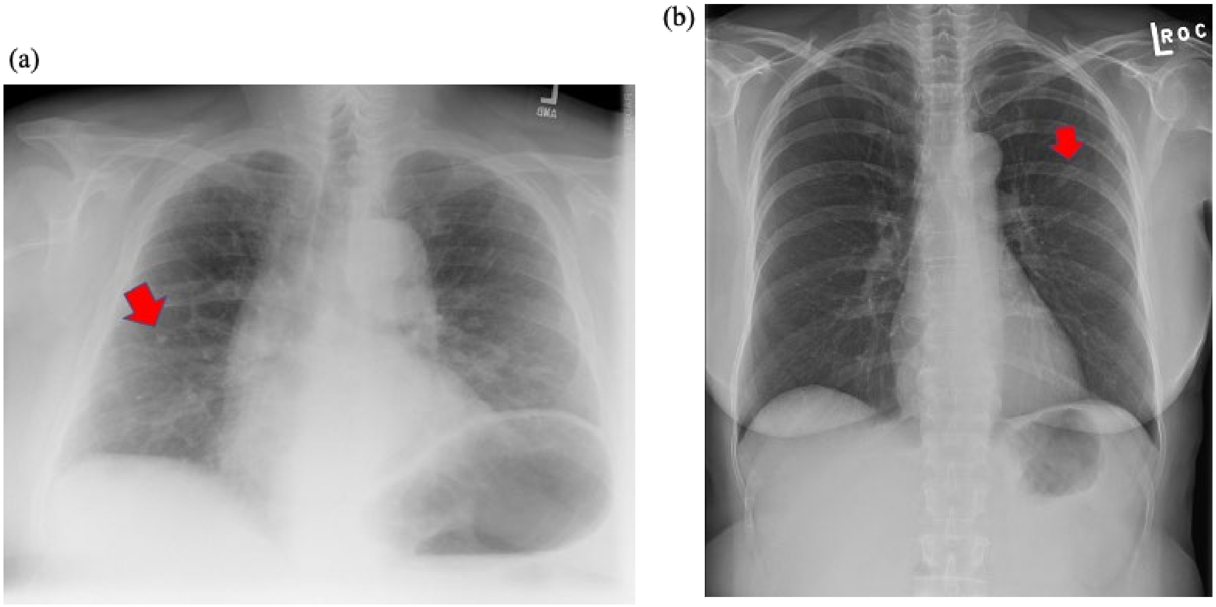

(a) CXR of a male in his 60s who was diagnosed with cancer 251 days after his baseline imaging. The AI missed a small nodule in the right middle lung zone (in red arrow). (b) CXR of a female in her 60s who was diagnosed with lung cancer 71 days after her baseline imaging. The AI missed a faint, oval nodule in the left upper lung zone (in red arrow), which was partly overlapping with the 7th rib.

Note. AI did not produce localization outputs for both CXR images as the abnormality score was less than the operating point.

© 2020 Yoo H et al. *JAMA Network Open*.
